# Study of the influence of introgression from chromosome 2 of the At subgenome of cotton Gossypium barbadense L. during backcrossing with the original lines of G. hirsutum L.

**DOI:** 10.18699/vjgb-25-125

**Published:** 2025-12

**Authors:** M.F. Sanamyan, Sh.U. Bobokhujayev, Sh.S. Abdukarimov, J.S. Uralov, A.B. Rustamov

**Affiliations:** National University of Uzbekistan named after Mirzo Ulugbek, Tashkent, Uzbekistan; National University of Uzbekistan named after Mirzo Ulugbek, Tashkent, Uzbekistan Chirchik State Pedagogical University, Chirchik, Tashkent region, Uzbekistan; National University of Uzbekistan named after Mirzo Ulugbek, Tashkent, Uzbekistan Center of Genomics and Bioinformatics of the Academy of Sciences of the Republic of Uzbekistan, Kibrai district, Tashkent region, Uzbekistan; National University of Uzbekistan named after Mirzo Ulugbek, Tashkent, Uzbekistan; National University of Uzbekistan named after Mirzo Ulugbek, Tashkent, Uzbekistan

**Keywords:** cotton, G. hirsutum, G. barbadense, monosomic lines, chromosome-substituted hybrids, backcrossing, SSR markers, хлопчатник, G. hirsutum, G. barbadense, моносомные линии, хромосомно-замещенные гибриды, беккроссирование, SSR-маркеры

## Abstract

The creation of chromosome substitution lines containing one pair of chromosomes from a related species is one method for introgression of alien genetic material. The frequency of substitutions in different chromosomes of the genome varies due to the selective transmission of alien chromosomes through the gametes of hybrids. The use of monosomic lines with identified univalent chromosomes and molecular genetic SSR markers at the seedling stage allowed rapid screening of the identity of the alien chromosome in backcross hybrids, significantly accelerating and facilitating the backcrossing process for the creation of new chromosome substitution cotton lines. As a result of studying the process of transmission of chromosome 2 of the At subgenome of the cotton plant G. barbadense L. during backcrossing of four original monosomic lines of G. hirsutum L. with monosomic backcross hybrids with substitution of chromosome 2 of the At subgenome, the following specific consequences of the introgression of this chromosome were revealed: decreased crossability, setting and germination of hybrid seeds; differences in the frequency and nature of transmission of chromosome 2 of the At subgenome of the cotton plant G. barbadensе; regularity of chromosome behavior in meiosis; a high meiotic index; a significant decrease in pollen fertility in backcross monosomic hybrids BC1F1; specific morphobiological characteristics of monosomic backcrossed plants, such as delayed development of vegetative and generative organs; dwarfism; reduced foliage; and poor budding and flowering during the first year of vegetation. All of these factors negatively impact the study and backcrossing of monosomic hybrids and significantly complicate and delay the creation of chromosome-substituted forms concerning chromosome 2 of the At subgenome of cotton, G. barbadense. These specific changes likely occurred as a result of hybrid genome reorganization and introgression of alien chromatin. Furthermore, the effectiveness of using molecular genetic microsatellite (SSR) markers to monitor backcrossing processes and eliminate genetic material from the Pima 3-79 donor line of G. barbadense for the selection of genotypes with alien chromosome substitutions has been demonstrated.

## Abstract (ru)

Создание хромосомно-замещенных линий, содержащих одну пару хромосом родственного вида, является одним из способов интрогрессии чужеродного генетического материала. Известно, что частота встречаемости замещений по разным хромосомам генома различается по причине избирательности процесса трансмиссии чужеродных хромосом через гаметы гибридов. Использование моносомных линий с идентифицированными унивалентными хромосомами и молекулярно-генетических SSR-маркеров на стадии проростков позволило осуществить быстрый скрининг идентичности чужеродной хромосомы у беккроссных гибридов, что значительно ускорило и облегчило процесс беккроссирования при создании новых хромосомно - замещенных линий хлопчатника. В ходе изучения процесса передачи хромосомы 2 Аt-субгенома хлопчатника Gossypium barbadense L. при беккроссировании четырех исходных моносомных линий вида G. hirsutum L. моносомными беккроссными гибридами с замещением хромосомы 2 Аt-субгенома были выявлены: снижение скрещиваемости, завязываемости и всхожести гибридных семян; отличия в частоте и характере передачи хромосомы 2 At-субгенома хлопчатника G. barbadensе; регулярность поведения хромосом в мейозе и высокий мейотический индекс; значительное снижение фертильности пыльцы у беккроссных моносомных гибридов BC1F1; специфические морфобиологические особенности моносомных беккроссных растений в виде задержки развития вегетативных и генеративных органов, низкорослости и сниженной облиственности, а также слабой бутонизации и цветения в течение первого года вегетации. По-видимому, такие специфические изменения происходили вследствие реорганизации гибридного генома и интрогрессии чужеродного хроматина. Кроме того, была продемонстрирована эффективность использования молекулярно-генетических микросателлитных (SSR) маркеров для контролирования процессов беккроссирования и элиминации генетического материала донорной линии Pima 3-79 вида G. barbadense с целью отбора генотипов с чужеродным замещением хромосом.

## Introduction

Currently, the lack of genetic diversity in the cultivated cotton plant Gossypium hirsutum L. (2n = 52, AD1) hinders the development of breeding programs (Wendel, 1989). One method of introducing alien genetic material is the creation of chromosome substitution lines containing one pair of chromosomes from a related species, which substitutes the homeologous pair of chromosomes because the substitution occurs in strict accordance with chromosome homeology (Shchapova, Kravtsova, 1990).

Lines with alien chromosome substitutions were created in the USA via three tetraploid cotton species (G. barbadense L., G. tomentosum Nutt. ex Seem and G. mustelinum Miers ex Watt) (Saha et al., 2004, 2006, 2013), where the largest number of lines were obtained from the G. barbadense species, and a study was conducted on the effects of substitution on valuable fibre quality traits (Saha et al., 2004, 2010, 2020; Jenkins et al., 2006, 2007). However, the active use of these lines by other researchers indicated the absence of introgression of the entire chromosome or a chromosomal region in some of these lines (Gutiérrez et al., 2009; Saha et al., 2015; Ulloa et al., 2016; Fang et al., 2023), so only 13 CS-B (chromosome substitution) lines out of 20 had “significant introgression” from the G. barbadense species. However, the reasons for the lack of substitution of alien chromosomes in the lines of the American collection have never been clarified.

Earlier, at the National University of Uzbekistan named after Mirzo Ulugbek a collection of new monosomic lines of G. hirsutum cotton was created via various irradiation methods. Univalent chromosomes of 35 of these lines, which are deficient in individual chromosomes, were identified via translocation and molecular genetic SSR markers (Sanamyan et al., 2014, 2022). Four monosomic lines were deficient in chromosome 2, 18 lines were deficient in chromosome 4, five lines were deficient in chromosome 6, one line was deficient in chromosome 7, and another line was deficient in chromosome 12 of the At subgenome of cotton. There is also one monosomic line on chromosomes 17, 18, 21 and 22 of the Dt subgenome of cotton and two telocentric lines on chromosomes 6 and 11 of the At subgenome (Sanamyan et al., 2016a, b; Sanamyan, Bobokhujaev, 2019).

To create chromosome substitution cotton lines, we developed a new scheme based on cytogenetic and molecular genetic methods (Sanamyan et al., 2022). The use of monosomic lines with identified univalent chromosomes and molecular genetic SSR markers at the seedling stage allowed rapid screening of the identity of alien chromosomes in backcross hybrids, significantly accelerating and facilitating the creation of chromosome substitution lines

Experiments involving many hybrids across multiple generations have revealed new effects of alien chromosome transmission in backcrossed progeny. The aim of our study was to analyse the transmission patterns of chromosome 2 of the At subgenome of cotton during backcrossing of four original monosomic lines of G. hirsutum with the donor line Pima 3-79 of G. barbadense to create G. hirsutum/G. barbadense lines with alien chromosome substitutions. We assessed the crossability, seed set, and germination of hybrid seeds, estimated the frequency and pattern of chromosome 2 transmission, studied chromosome behavior during meiosis, and identified specific morphobiological features of backcrossed monosomic plants in the first year of vegetative growth.

## Materials and methods

Plant material. The cotton cytogenetic collection of the National University of Uzbekistan named after M. Ulugbek is characterized by the presence of four monosomic lines (Mo11, Mo16, Mo19, and Mo93) deficient in chromosome 2 of the At subgenome of the tetraploid cotton species G. hirsutum (2n = 52, AD1) (Sanamyan, Bobokhujaev, 2019).

The Pima 3-79 line, which was obtained from a doubled haploid and is the genetic standard for this cotton species in the United States (Endrizzi et al., 1985), was used as the donor parent of the substitution chromosome (CS) from the G. barbadense species.

Backcross hybrids obtained from crossing four monosomic lines (Mo11, Mo16, Mo19, and Mo93) deficient in chromosome 2 of the At subgenome of the G. hirsutum species with previously obtained monosomic F1 hybrids, which had monosomy for the same chromosomes as the original monosomic plants (Sanamyan et al., 2016b), were studied. All plants of the original monosomic lines and backcross hybrids were maintained year-round in a film greenhouse at the National University of Uzbekistan.

Cytological analyses, as well as DNA extraction and genotyping, were performed according to methods described previously (Sanamyan et al., 2023). Elimination of G. barbadense chromosomes in the monosomic cotton hybrid BC2F1 was determined by the absence of marker amplification on the G. barbadense chromosomes (paternal) and the presence of only allele-specific PCR products of G. hirsutum (maternal).

## Results

Crossability, seed set, and germination of BC1F1 hybrid seeds involving monosomic lines deficient in chromosome 2 of the At subgenome of G. hirsutum. Four monosomic lines of G. hirsutum deficient in chromosome 2 of the At subgenome were crossed with F1 hybrids (Mo×Pima 3-79), which were monosomic for the same chromosomes as the original monosomic line. All four BC1F1 variants were characterized by a significant decrease in the percentage of crossability (from 33.33 to 9.09 %) (Supplementary Material 1)1 compared with F1 hybrids (from 68.75 to 50.00 %) (Sanamyan et al., 2022).


Supplementary Materials are available in the online version of the paper: https://vavilovj-icg.ru/download/pict-2025-29/appx45.pdf


The hybrid seed set of the BC1F1 plants also decreased (from 52.94 ± 12.11 to 37.31 ± 5.91 %) compared with that of the F1 hybrids (from 57.69 ± 9.69 to 31.33 ± 5.09 %). Compared with that of the F1 hybrids, the germination of the backcrossed BC1F1 seeds decreased from 88.89 to 51.61 % (Supplementary Material 1) (from 100 to 71.43 %).

Identification of chromosome 2 substitutions in the At subgenome of G. barbadensе in BC1F1 hybrids via chromosome-specific molecular genetic markers. For molecular analysis of backcrossed BC1F1 plants, the principles of deletion molecular analysis were used (Liu et al., 2000; Gutiérrez et al., 2009). The study was conducted according to our previously proposed scheme for producing chromosome substitution cotton lines (Sanamyan et al., 2022). Molecular genetic analysis of the backcrossed forms was performed on backcrossed cotton seedlings at the 3–5 true leaf stage before they were transplanted into greenhouse soil to accelerate the isolation of monosomic chromosomes from the donor species. Among the plants harboring all the BC1F1 variants, those with genomes containing an alien chromosome 2 of the At subgenome of the G. barbadense species were identified.

Previously, among the BC1F1 hybrids in the BC1F1(Mo11× ×F1(7663)) cross variant in four backcross families (9n, 10n, 78n, and 79n), no hybrid seedlings with polymorphic alleles from the G. barbadense species were found, which indicated the absence of substitutions in these monosomes. Later, in a similar variant of the experiment in the backcross family (494n), one (4942) monosomic backcross seedling was identified, which was characterized by the presence of polymorphic alleles only from G. barbadense, whereas the alleles of the L-458 line of the G. hirsutum species were not detected on the basis of the localization of chromosome-specific SSR markers, BNL834, BNL1434, BNL1897, BNL3590, BNL3971, BNL3972, CIR381, and JESPR179. Since all the above-mentioned markers were previously localized on chromosome 2 of the At subgenome of cotton (Liu et al., 2000; Gutiérrez et al., 2009; Yu et al., 2011; Saha et al., 2015; Wang et al., 2016), the obtained data indicated the presence of substitutions on this chromosome (Supplementary Materials 2, 3, 19).

Previously, in the BC1F1(Mo16×F1(986)) variant, four seedlings (9222, 9228, 9237, 9238) with a substitution on chromosome 2 of the G. barbadense species were already found in two hybrid backcross families (922n and 923n) (Sanamyan et al., 2022); however, the elimination of the alien chromosome in BC2F1 required re-examination of this variant, where three backcross seedlings (4955, 4971 and 4974) were found in two new families, which had only alleles from G. barbadense, whereas the alleles of G. hirsutum were absent, which indicated the localization of seven chromosome-specific SSR markers: BNL834, TMB0471, JESPR101, JESPR179, CIR376, DPL0674, and NAU2277, previously localized on chromosome 2 of the At subgenome (Gutiérrez et al., 2009; Yu et al., 2011; Saha et al., 2015; Wang et al., 2016) and confirmed the presence of a substitution on this chromosome (Supplementary Materials 4–6, 19).

**Fig. 1.**
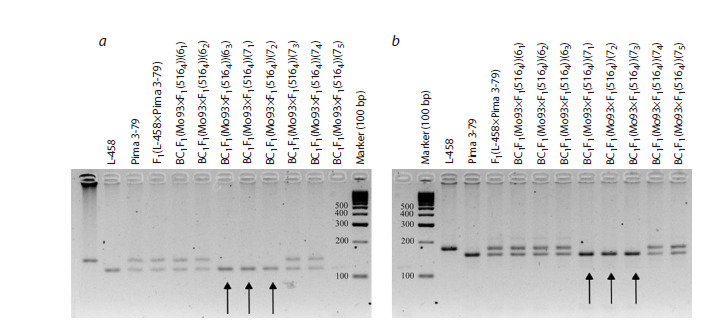
Electropherogram of DNA amplicons of SSR markers in hybrid seedlings BC1F1(Mо93×F1(5164)) on chromosome 2 of the At subgenome of cotton. a – BNL3971; b – JESPR179.

SSR-based deletion analysis in the BC1F1(Mo19×F1(7694)) and BC1F1(Mo93×F1(5164)) combinations with a putative substitution of chromosome 2 of the At subgenome of cotton allowed us to detect alleles of the G. barbadense species in one backcross seedling (88) in the first variant and nine seedlings (71, 72, 73, 881, 884, 887, 893, 894 and 896) in the second, whereas alleles of the G. hirsutum species were absent. Since chromosome-specific SSR markers BNL3545, BNL3971, JESPR101, JESPR179, CIR376, and DPL0674 were previously localized on chromosome 2 of the At subgenome of cotton (Liu et al., 2000; Gutiérrez et al., 2009; Yu et al., 2011; Saha et al., 2015; Wang et al., 2016), the obtained data indicated the presence of substitution of this chromosome in the studied seedlings (Fig. 1, Supplementary Materials 7–9, 19).

A study of meiosis in BC1F1 hybrids with identified univalents. Chromosome pairing at the metaphase I (MI) stage of meiosis was studied in 17 monosomic plants in four backcross variants obtained from crosses of monosomic lines of the G. hirsutum species with interspecific monosomic F1 hybrids (Mo×Pima 3-79). Seven monosomics were found among the backcrossed plants in the BC1F1(Mo16×F1(986)) variant, one each in the BC1F1(Mo11×F1(7663)) and BC1F1(Mo19×F1(7694)) variants, and eight in the BC1F1(Mo93×F1(5164)) variant.

**Fig. 2.**
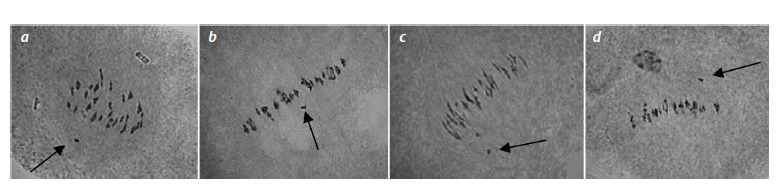
Chromosome configurations in metaphase I of meiosis in BC1F1 hybrid plants obtained from crossing monosomic lines with interspecific monosomic F1 hybrids: F1: a – BC1F1(Мо11×F1(7663)) (4942); b – BC1F1(Mo16×F1(982)) (4971); c – ВС1F1(Мо19×F1(7694)) (88); d – ВС1F1(Мо93×F1(5164)) (881) with chromosome 2 of the At subgenome of G. barbadense. Univalents are indicated by arrows.

Analysis of meiotic MI in BC1F1 monosomic plants, where all backcrossed monosomic plants had univalent G. barbadense chromosomes, revealed a modal chromosome pairing with 25 bivalents and one univalent chromosome, characteristic of tetraploid monosomic cotton plants (Supplementary Material 10). Analysis of the size of univalents in monosomic BC1F1 confirmed the large size of chromosome 2 of G. barbadense in all four crossing variants (Fig. 2), which indicated that this chromosome belongs to the At subgenome of cotton.

**Fig. 3.**
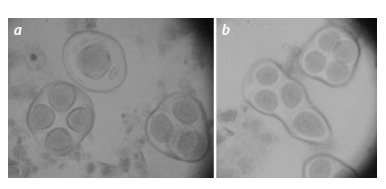
Sporades in the hybrid plant BC1F1(Мо93×F1(5164)) (887): a – monad with three micronuclei and tetrads; b – abnormal tetrad and normal tetrad.

Most of the studied ВС1F1 monosomics were characterized by a high meiotic index (up to 95.74 ± 0.47) and a small number of tetrads with micronuclei (up to 3.86 ± 0.65 %), with the exception of two monosomics (9228 and 73) with a reduced meiotic index (89.04 ± 0.94 and 88.57 ± 1.13, respectively) and an increased number of tetrads with micronuclei (up to 4.75 ± 0.64 and 5.15 ± 0.78 %, respectively) (Fig. 3, Supplementary Material 11).

**Fig. 4.**
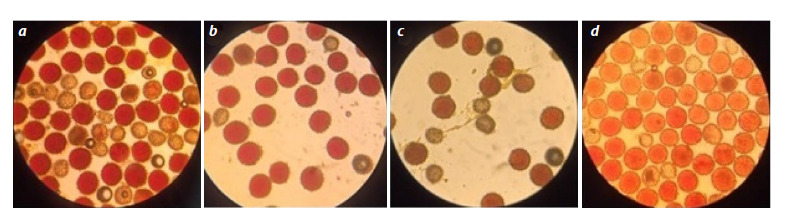
Sterile (uncolored) and fertile (colored) pollen in monosomic BC1F1 hybrids obtained from crossing a monosomic line with a monosomic hybrid F1(Мо×Pima 3-79): a, b (884) and c, d (894) in the BC1F1(Mo93×F1(5164)) variant.

Pollen viability was assessed in BС1F1 a b monosomics via acetocarmine staining. Only two monosomic strains (9228 and 9237) presented high pollen viability (up to 94.21 ± 1.06 %) (Fig. 4, Supplementary Material 12). Six monosomics methods resulted in a slight reduction in pollen viability (from 88.38 ± 1.46 to 81.15 ± 1.52 %), but five monosomics methods (72, 881, 884, 887, and 894) involving one variant of BC1F1 (Mo93×F1(5164)) were characterized by a strong reduction in pollen viability (from 62.65 ± 2.15 to 77.47 ± 1.64 %).

Crossability, seed set, and germination characteristics of BC2F1 hybrid seeds with monosomic lines deficient in chromosome 2 of the At subgenome of G. hirsutum. Three monosomic lines deficient in chromosome 2 of the At subgenome of G. hirsutum from the cytogenetic collection were crossed with monosomic BC1F1(Мо×F1(Мо×Pima 3-79)) hybrids that contained substitutions of chromosome 2 of the At subgenome of G. barbadense. Compared with those of the F1 and BC1F1 hybrids, a strong decrease in crossability was observed (from 18.18 to 3.47 %) (Supplementary Material 13). A study of the seed set rate of hybrid BC2F1 plants revealed a significant decrease (from 47.47 ± 5.02 to 27.69 ± 5.55 %) compared with that of the F1 and BC1F1 hybrids, while the germination rate of backcrossed BC2F1 seeds also decreased (from 69.23 to 44.44 %) compared with that of the same hybrids, with the monosomic line Mo93 exhibiting the strongest decrease in seed set and germination compared with the other two monosomic lines.

Identification of substitutions in the chromosome 2 of the At subgenome of G. barbadense in BC2F1 and BC3F1 hybrids via chromosome-specific molecular genetic markers. We previously demonstrated that five monosomic seedlings (211, 212, 214, 217 and 221) in the BC2F1 (Mo16×BC1F1(9237)) variant with a putative substitution of chromosome 2 in the At subgenome of cotton were characterized by the presence of chromosome-specific alleles only from the L-458 line of G. hirsutum, whereas alleles from G. barbadense were absent, indicating the elimination of the alien chromosome (Sanamyan et al., 2023).

**Fig. 5.**
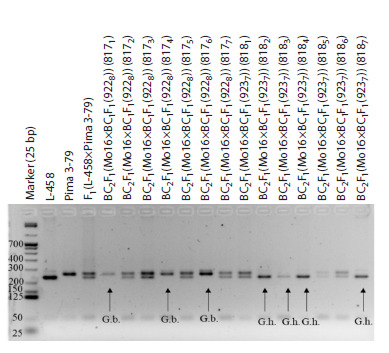
Electropherogram of DNA amplicons of SSR markers in hybrid seedlings, BC2F1(Mо16×BC1F1(9228)) on chromosome 2 of the At subgenome of cotton. Marker DPL0674.

The results of a new study of two variants involving the Mo16 line but two different BC1F1 hybrids (BC2F1(Mo16× ×BC1F1(9228)) and BC2F1(Mo16×BC1F1(9237)) revealed that in the first variant, three seedlings (8171, 8174 and 8176) had chromosome-specific alleles only from the G. barbadense species, whereas alleles from the G. hirsutum species were absent; however, in the second variant, four seedlings (8182, 8183, 8184 and 8187) had alleles only from the G. hirsutum species, which indicated the elimination of the alien chromosome. Since previously reported chromosome-specific SSR markers, BNL3971, TMB1194, CIR376, and DPL0674 are located on chromosome 2 of the At subgenome of cotton (Liu et al., 2000; Gutiérrez et al., 2009; Saha et al., 2013, 2015; Wang et al., 2016), the obtained data indicate the presence of chromosome 2 substitutions in the first three seedlings (Fig. 5, Supplementary Materials 14, 19).

**Fig. 6.**
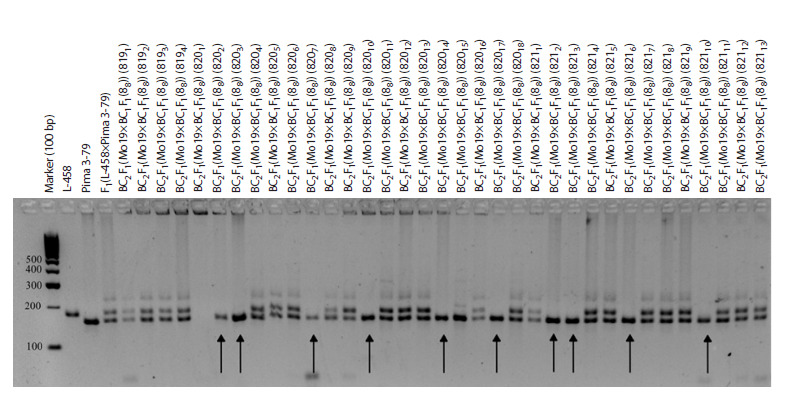
Electropherogram of DNA amplicons of SSR markers in hybrid seedlings, BC2F1(Мо19×BC1F1(88)) on chromosome 2 of the At subgenome of cotton. Marker JESPR179.

**Fig. 7.**
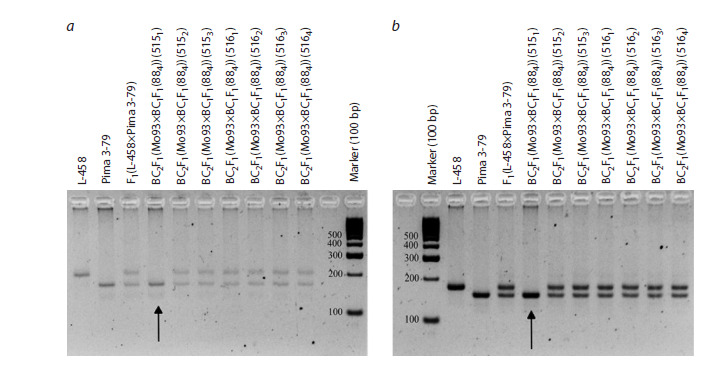
Electropherogram of DNA amplicons of SSR markers in hybrid seedlings, BC2F1(Mo93×BC1F1(884)) on chromosome 2 of the At subgenome of cotton. a – marker TMB0471; b – marker JESPR179.

SSR-based deletion analysis in combinations BC2F1(Mo19× ×BC1F1(88)) and BC2F1(Mo93×BC1F1(884)) with putative substitution of chromosome 2 of the At subgenome of cotton allowed us to detect alleles of the G. barbadense species only in 10 backcross seedlings (8202, 8203, 8207, 82010, 82014, 82017, 8212, 8213, 8216, and 82110) of the first variant and in one (5151) of the second, whereas alleles of the G. hirsutum species were absent. Chromosome-specific SSR markers, such as BNL834, BNL1434, BNL1897, BNL3292, BNL3413, BNL3424, BNL3547, BNL3971, BNL3972, JESPR101, JESPR179, JESPR304, TMB0471, TMB1194, CIR376, CIR381, CIR401, DPL0674, and NAU2277 are located on chromosome 2 of the At subgenome of cotton (Liu et al., 2000; Gutiérrez et al., 2009; Saha et al., 2013, 2015; Wang et al., 2016; https://www.CottonGenorg/data/download/ marker_origin), and the obtained data indicated the presence of substitutions of this chromosome in the studied seedlings (Fig. 6, 7, Supplementary Materials 15–17, 19)

**Fig. 8.**
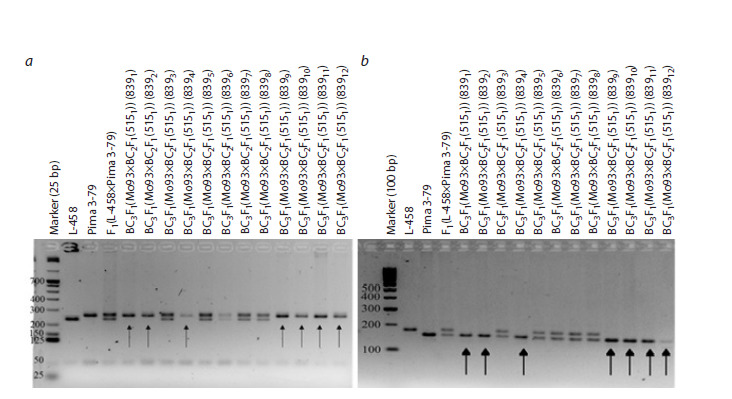
Electropherogram of DNA amplicons of SSR markers in hybrid seedlings, BC3F1(Mo93×BC2F1(5151)) on chromosome 2 of the At subgenome of cotton. a – marker DPL0674; b – marker JESPR179.

Similarly, in the BC3F1(Mo93×BC2F1(5151)) combination with a putative substitution of chromosome 2 of the At subgenome of cotton, alleles of G. barbadense were detected in seven backcrossed seedlings (8391, 8392, 8394, 8399, 83910, 83911, and 83912), whereas alleles of the G. hirsutum species were absent. Because previously reported chromosome-specific SSR markers, DPL0674 and JESPR179, were located on chromosome 2 of the At subgenome of cotton (Liu et al., 2000; Gutiérrez et al, 2009; Yu et al., 2011; Saha et al., 2013, 2015; Wang et al., 2016), the obtained data indicated the presence of a substitution of this chromosome in the studied seedlings (Fig. 8, Supplementary Materials 18, 19).

Study of meiosis in BC2F1 hybrids with identified univalent. A study of chromosome pairing at the MI stage of meiosis revealed one backcrossing monosomic in each of the three backcrossing variants of BC2F1 (involving the Mo16, Mo19, and Mo93 lines) at the time of writing.

Analysis of meiotic MI in three BC2F1 monosomic strains, including one monosomic strain, 211, from one cross with univalent chromosome 2 of the At subgenome of cotton G. hirsutum (Sanamyan et al., 2023) and two other monosomic strains (82110 and 5151) from two crosses with univalent chromosomes 2 of G. barbadense, revealed modal pairing of chromosomes with 25 bivalents and one univalent, confirming their monosomic status. Univalent size analysis in BC2F1 monosomics revealed a large chromosome 2 of G. hirsutum and a similar size in BC2F1 monosomic data with chromosome 2 of G. barbadense

**Fig. 9.**
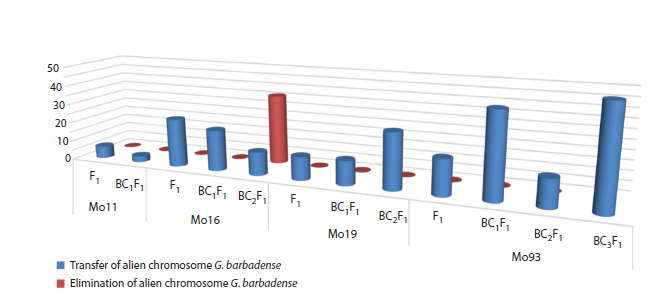
Frequency of occurrence of monosomic hybrids with alien chromosome 2 of the At subgenome of cotton G. barbadense.

Characteristics of the transmission of chromosome 2 of the At subgenome of cotton during backcrossing of monosomic lines deficient in this chromosome by monosomic hybrids of different backcross generations. Molecular genetic analysis of backcrossed seedlings (at the stage of 3–5 true leaves), carried out via chromosome-specific molecular markers (SSR), allowed us to estimate the frequency and nature of the transmission of chromosome 2 from the At subgenome of cotton in BC1F1 hybrid plants. A study of five backcrossed BC1F1 families revealed that, in the BC1F1(Mo11×F1(7663)) variant, there was only one seedling (4942) with the presence of the substituted chromosome, which indicated a very rare transmission frequency of chromosome 2 of the At subgenome of the G. barbadense species (3.13 %) (Fig. 9, Supplementary Material 20).

Among the BC1F1 hybrids in the BC1F1(Mo16×F1(986)) cross combination, seven seedlings with chromosome 2 substitutions were found, which were characterized by the presence of polymorphic alleles only from the G. barbadense species, indicating a relatively high frequency of transmis-sion of chromosome 2 from the At subgenome of the G. barbadense species (21.21 %) in this variant. However, a study of the transmission of this chromosome in BC2F1 hybrids in the BC2F1(Mo16×BC1F1(9237)) variant revealed the presence of chromosome-specific alleles only from the G. hirsutum species, whereas alleles from the G. barbadense species were absent, which revealed the absence of chromosome 2 substitution in five hybrids studied. Repeated analysis of BC2F1 hybrids in two cross variants allowed us to identify four hybrids in one BC2F1(Mo16×BC1F1(9237)) variant with chromosome-specific alleles only from the G. hirsutum species, which drew attention to the repeated elimination of this chromosome, whereas in the other variant (9228), the presence of chromosome 2 substitution from the G. barbadense species was observed. In general, in BC2F1 with the participation of the monosomic line Mo16, the frequency of transmission of the substituted chromosome was characterized by a decrease (up to 12.00 %) compared with that in BC1F1 hybrids, as well as elimination in the majority of hybrids (36.00 %). Moreover, elimination of the substituted chromosome occurred in one variant of crosses involving the same BC1F1 hybrid plant, which suggested the influence of genotype on the nature of chromosome transmission (Fig. 9, Supplementary Material 20).

Among the hybrids of one family in the BC1F1(Mo19×F1(7694)) variant with a putative chromosome 2 substitution, a single hybrid plant (88) was identified that had an allele from G. barbadense, which indicated the transmission of the substituted chromosome 2 with a low frequency (12.50 %). However, when the transmission of this chromosome in BC2F1 hybrids in the BC2F1(Mo19×BC1F1(88)) variant was studied, chromosome-specific alleles from the G. barbadense species were detected only in ten hybrids in three analysed families, which indicates greater transmission of the substituted chromosome 2 (28.57 %) than in BC1F1 hybrids (Fig. 9, Supplementary Material 20).

Nine hybrids with chromosome 2 substitutions were detected in the BC1F1(Mo93×F1(5164)) variant, which was characterized by the presence of alleles from G. barbadense only, indicating a high frequency of transmission of this chromosome (42.86 %). Among the BC2F1 hybrids in the BC2F1(Mo93×BC1F1(884)) variant, only one hybrid from two families had a substitution from G. barbadense, confirming the low frequency of chromosome 2 substitution (14.29 %). However, the study of the transmission of this chromosome in BC3F1 hybrids in the BC3F1(Mo93×BC2F1(5151)) variant made it possible to characterize the presence of chromosomespecific alleles from the G. barbadense species in seven hybrids out of 12 studied, i. e., the transmission of chromosome 2 was observed in more than half of the hybrids (58.33 %) (Fig. 9, Supplementary Material 20).

**Fig. 10.**
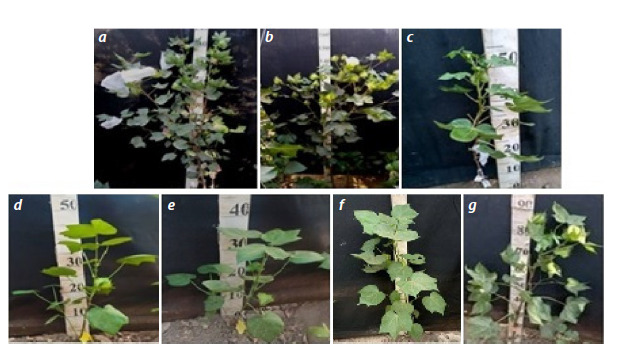
Plants of the original lines and monosomic hybrids of cotton F1 and BC2F1 obtained from crossings of the recurrent parent with monosomic hybrids. a – monosomic line Mo16 for chromosome 2 of the At subgenome; b – F1(Мо16×Pima 3-79) (982); c–e – BC1F1(Mo16×F1986) with substitution of chromosome 2 of the At subgenome of cotton G. barbadense: c – 9237, d – 4955, e – 4974; f – BC2F1(Mo16×BC1F19228) (8174) with substitution of chromosome 2; g – BC2F1(Mo16×BC1F19237) (211) without substitution of chromosome 2 of the At subgenome of cotton G. barbadense

Morphobiological analysis of monosomic hybrids BC1F1, BC2F1, and BC3F1 obtained from crosses of the monosomic cotton lines G. hirsutum with chromosome 2 deficiency of the At subgenome with monosomic hybrids of different generations of backcrosses with chromosome substitution. Morphobiological analysis of backcross monosomic hybrids BC1F1 with chromosome 2 substitution of the At subgenome of cotton revealed short stature (up to 50 cm on average); reduced size of three-lobed leaves, buds, and flowers; low foliage; shortened internodes; and weak budding and flowering during the first year of vegetation (only 2–3 buds and flowers per plant) compared with the recurrent parent. The backcross hybrids with the participation of the monosomic line Mo16 (Fig. 10) were characterized by particularly short stature and a decrease in growth rates, whereas in the second and third years of vegetation, there was an increase in the size of the leaves, the number of buds and the number of opened bolls.

Compared with the monosomic hybrid BC1F1, with the substitution of chromosome 2, the complemented hybrids ВС2F1 and ВС3F1, with the substitution of chromosome 2 of the At subgenome of cotton, were distinguished by taller growth (up to 80 cm), medium foliage, larger three-lobed leaves, an increased number of flowers and medium-sized spherical bolls.

## Discussion

Interspecific crosses of various tetraploid cotton species are characterized by high crossability and fertility of firstgeneration hybrids. However, in later generations, decreases in all these parameters and mass sterility are observed (Mauer, 1954; Abdullaev, 1974).

A study of the crossability and seed set of backcrossed seeds obtained from crosses of monosomic lines deficient in chromosome 2 of the At subgenome of cotton G. hirsutum with interspecific monosomic F1 hybrids and backcrossed monosomic ВС1F1 hybrids revealed a significant decrease in crossability in all the studied backcrossed hybrids, whereas hybrid seed set and germination decreased in most hybrids. This decrease was explained by the decrease in these parameters in the original monosomic lines due to the presence of a significant number of nullisomic gametes, as well as the hemizygosity of the maternal and paternal plants, which resulted in the presence of numerous unfertilized eggs in the form of motes and low germination of nullisomic seeds.

Molecular genetic methods using microsatellite sequence markers (SSRs), which are widespread in eukaryotic genomes and exhibit a high level of polymorphism, are widely used to identify alien introgression. To date, several collections of such markers have been created for cotton (BNL, JESPR, CIR, DPL, and NAU), and molecular genetic linkage maps have been constructed due to chromosomal specificity of many SSR markers (Liu et al., 2000; Gutiérrez et al., 2009; Yu et al., 2011; Saha et al., 2013, 2015; Wang et al., 2016; https://www. cottongen.org/data/download/ marker_origin). The presence or absence of microsatellite marker amplification products after polymerase chain reaction allows us to judge the presence or absence of an alien chromosome in the studied genotype. Therefore, our molecular genetic screening of backcross hybrids at the seedling stage allowed us to quickly identify the substitution of chromosome 2 of the At subgenome of cotton of the G. barbadense species.

Compared with direct cytological observation, PCR-based screening for chromosome-specific markers has been shown to be more productive and significantly more effective (Polgári et al., 2019). This is especially true when it is impossible to detect and monitor alien genetic material directly on cytological preparations via FISH and GISH hybridization

The originality of our approach lies in the development of a new scheme for the targeted, planned substitution of specific chromosomes via monosomic lines with previously established univalent chromosome identities and chromosomespecific microsatellite markers (SSRs) at the seedling stage. During the creation of new chromosome substitution lines via this scheme, significant differences were identified between specific chromosomes in the cotton genome in terms of the frequency of transmission of alien chromosomes, their elimination at different stages of backcrossing, and the morphobiological characteristics of the backcrossed hybrids. This study presents data on the specific consequences of the transfer of genetic material from chromosome 2 of the At subgenome of cotton G. barbadense into the genome of cotton G. hirsutum via four monosomic lines.

The results of the study of backcross progenies BC1F1, BC2F1 and BC3F1 (in one variant of crossing) revealed that the frequency of transmission of chromosome 2 of the At subgenome of cotton, G. barbadense depended on both the genotype of the monosomic line and the genotype of the backcross hybrid used in the crossing. Thus, the level of transmission of chromosome 2 of the At subgenome of G. barbadense in the first backcross generation, with the participation of four monosomic lines, varied within a wide range (from 3.13 to 42.86 %), whereas in the BC2F1 generation, where only three monosomic lines were involved, the transmission frequency was noticeably reduced and varied within a narrower range (from 12.00 to 28.57 %). However, in the BC3F1 generation, which included only one monosomic line (Mo93), the highest frequency of chromosome 2 transmission (58.33 %) was observed in more than half of the studied hybrids. Moreover, this line was characterized by the highest average number of substitutions per hybrid genome among all four studied lines. In addition, the elimination of chromosome 2 of the At subgenome was detected in the BC2F1 variant (Mo16×BC1F1 (9237)), which involved the same paternal backcross monosomic hybrid plant (9237) during two experiments, indicating the preferential elimination of chromosome 2 in the studied backcross progenies and the influence of a specific paternal genotype on this process. Therefore, the analysis of the transmission characteristics of alien chromosome 2 revealed differences in the competitiveness of this chromosome when it is transmitted to offspring in the genotypic environment of four monosomic lines.

The success of substitution depends on how well the alien chromosome compensates for the missing chromosome, since it is difficult to assume that each alien chromosome could compensate equally for the absence of homeologous chromosomes (Morris, Sears, 1970).

Previously, a dependence of rye chromosome introgression on the genotypic environment of the recipient was discovered, since differences in the frequency of rye chromosomes were observed in the F2 hybrid population between a wheat-rye substitution line for chromosomes 1R+2R and the winter wheat varieties Holme and Kraka (Merker, Forsstrom, 2000). Notably, the genotype of the variety also influenced the frequency of telocentric formation on the rye chromosome and T2R.2DL translocation (Krasilova et al., 2011).

Cases of complete uniparental elimination of chromosomes from the entire genome are widely known (Ishii et al., 2016; Dedukh, Krasikova, 2021). They result from hybridization between distantly related species as an element of protecting the integrity of the genome from “genomic shock” (McClintock, 1984). An example is the preferential elimination of chromosomes from the entire D genome in first-generation hybrids during wheat-rye hybridization (Li et al., 2015).

Examples of partial elimination of individual chromosomes are less numerous, but it is known that in each specific case, different mechanisms of chromosome elimination operate in interspecific hybrids, depending on the specific species involved. Thus, an assessment of a large population of wheatbarley hybrids via genomic in situ hybridization (GISH) and simple sequence repeat (SSR) markers revealed the absence of preference for the elimination of individual barley chromosomes compared with wheat chromosomes (Polgári et al., 2019). A study of the transmission of chromosome 5R through the gametes of wheat-rye dimonosomics 5R5D revealed a significantly lower competitive ability of this chromosome in transmission to offspring and its preferential elimination (Silkova et al., 2011). Selective elimination of chromosomes in Hordeum bulbosum L. is associated with the loss of one of the types of histone protein H3 (CENH3) in the centromere, leading to its inactivity and absence of chromosome attachment to the mitotic spindle, as well as the formation of micronuclei and their degeneration (Sanei et al., 2011). A comparative analysis of the nucleotide sequences of the centromeric histone CENH3 genes in wheat-rye allopolyploids of different ploidy levels revealed increased expression of rye CENH3 variants, which is associated with the maintenance of a viable hybrid genome (Evtushenko et al., 2019). Using bread wheat as an example, a wide range of features of alien chromatin introgression was demonstrated, which represents significant potential for gene pool enrichment (Adonina et al., 2021).

Although screening for the presence of alien chromosomes in backcrossed cotton progenies via molecular genetic markers made it possible to detect specific consequences of the introgression of chromosome 2 of the At subgenome of the G. barbadense species at different stages of backcrossing, the study of the behavior of univalent chromosomes at the MI stage of meiosis revealed the similarity of pairing in backcrossed monosomes with the univalent chromosome 2 of the At subgenome of the cotton species G. hirsutum and the univalent chromosome 2 of the At subgenome of the G. barbadense species.

The backcrossed BC1F1 monosomic strains we examined were characterized by a general delay in the development of vegetative and generative organs, manifested by stunted growth, reduced foliage, and poor budding and flowering during the first year of vegetative development. This hampered their study and backcrossing and significantly complicated and delayed the creation of chromosome substitution forms. However, during the second year of vegetative development, the backcrossed plants showed normalization of vegetative and generative organ development.

Similar dwarfism was observed in the monosomic alien complemented cotton line G. hirsutum/G. bickii Proch. with a 10 Gb chromosome substitution (MAAL), created through crosses with an amphidiploid (2n = 78, AADDG1G1) and chromosome-specific SSR markers (Tang et al., 2018). It can be assumed that such changes in morphobiology occurred due to hybrid genome reorganization and introgression of alien chromatin.

## Conclusion

This study demonstrated the negative consequences of the introgression of chromosome 2 of the At subgenome of cotton, G. barbadense, into the genome of cotton, G. hirsutum, involving four monosomic lines. These negative consequences include decreased crossability, seed set, and germination of hybrid seeds; a wide variation in the transmission level of alien chromosome 2 (from 3.13 to 42.86 %) in BC1F1, but a less narrow one (from 12.00 to 28.57 %) in BC2F1; elimination of chromosome 2 of the At subgenome of the G. barbadense species in the BC2F1(Mo16×BC1F1(9237)) variant with the same paternal genotype, which indicated the influence of a specific paternal genotype on this process; dwarfism; and reduced foliage, weak budding and flowering in the first year of vegetative development but an increase in the number of buds, flowers and bolls in monosomic hybrids of the following backcross generations.

## Conflict of interest

The authors declare no conflict of interest.
